# Improvement of *in vitro* fertilization by a tannin rich vegetal extract addition to frozen thawed boar sperm

**DOI:** 10.21451/1984-3143-AR2019-0130

**Published:** 2020-05-06

**Authors:** Giovanna Galeati, Diego Bucci, Chiara Nerozzi, Beatrice Gadani, Carlo Tamanini, Beatrice Mislei, Marcella Spinaci

**Affiliations:** 1 Department of Veterinary Medical Sciences, University of Bologna, Ozzano dell’Emilia, BO, Italy; 2 AUB-INFA National Institute of Artificial Insemination, Bologna, Cadriano, BO, Italy

**Keywords:** pig, frozen semen, polyphenols, fertilization

## Abstract

Boar spermatozoa are very susceptible to cryopreservation injuries and, for this reason, pig remains one of the few species in which fresh semen is still preferred to thawed one for routine artificial insemination (AI). The present work evaluated the effect of supplementing boar sperm thawing medium with Silvafeed SP (SSP), a mixture of Chestnut and Quebracho wood extracts (60/40 w/w) rich in polyphenols (92.4% tannin content) on *in vitro* fertilization (IVF) and on the following sperm parameters: sperm motility (assessed by CASA), viability, acrosome integrity, mitochondrial function and lipid peroxidation (assessed by flow cytometry) and capacitation status (immunolocalization of tyrosine phosphorylated proteins). Thawed spermatozoa were incubated 1 h at 37°C in BTS without (CTR) or with (5, 10, 20 µg/mL) SSP. After incubation sperm suspension was divided in three aliquots: one was used for IVF trials, one for sperm analysis, and the last one was capacitated for 1 h at 39°C 5% CO_2_ in IVF medium. Sperm motility parameters, viability, acrosome integrity, mitochondrial functionality, lipid peroxidation and tyrosine phosphorylated protein immunolocalization, used as capacitation parameter, were not influenced by SSP. However, oocytes inseminated with thawed spermatozoa pretreated with all the different SSP concentrations presented a significant (P < 0.01) increase in penetration rate compared to CTR. In addition, 5 µg/mL SSP exerted a positive effect (P<0.05) on the total efficiency of fertilization. These results encourage the use of SSP in the thawing medium since post-thawing fertility is a limit for the large-scale use of boar frozen semen.

## Introduction

Artificial insemination (AI) with frozen-thawed semen in commercial swine herds is limited due to the low fertility outcomes compared to extended fresh semen (Johnson et al., 2000; Knox, 2015, 2016; Yeste et al., 2017). The low fertility is largely due to the reduction of fertilizing ability of spermatozoa during the cryopreservation process. In fact, approximately 50% of the spermatozoa in an ejaculate survive the current freezing and thawing process (Johnson et al., 2000; Bailey et al., 2008). It is well known that boar spermatozoa are very susceptible to low temperature, and therefore to cryopreservation process, due to their membrane structure rich in polyunsaturated fatty acids (PuFAs) and poor in cholesterol molecules (Rooke et al., 2001). During cryopreservation, cold shock leads the sperm plasma membrane to destabilize affecting acrosome integrity, membrane lipid packaging and inducing the loss of fertilizing potential (Yeste, 2015). Moreover, spermatic cells enter a stage of metabolic stress called “oxidative stress” characterized by a high production of Reactive Oxygen Species (ROS) and by a decrease of the antioxidant defenses due to seminal plasma removal during sperm preparation for freezing procedure (Guthrie and Welch, 2012).

The bad tolerance of boar semen to cryopreservation represents a considerable limit for the routine field application in o a wide scale and pig remains one of the few species in which fresh semen is still preferred to thawed one for insemination. Therefore, numerous researchers have evaluated the effects of supplementing frozen and/or thawing media with a wide variability of antioxidants in order to increase the percentage of viable cells after storage and to enhance their quality and fertilizing ability (Roca et al., 2004; Kaeoket et al., 2010; Yeste, 2015; Giaretta et al., 2015; Gadani et al., 2017; Bucci et al., 2018).

As recent studies demonstrated that supplementation of the thawing medium of boar frozen semen with natural polyphenols (epigallo-catechin-3-gallate and resveratrol) positively affects *in vitro* fertilization parameters (Gadani et al., 2017; Bucci et al., 2018), the aim of the present work was to study the effect of supplementing boar sperm thawing medium with Silvafeed SP (SSP) (blend of tannins) on different sperm parameters: motility (assessed by CASA), viability, acrosome integrity, mitochondrial function and lipid peroxidation (assessed by flow cytometry) and capacitation state (assessed by immunolocalization of tyrosine-phosphorylated proteins). A further objective was to determine the influence of SSP on *in vitro* fertilization (IVF).

## Materials and methods

Unless otherwise specified, all chemicals were purchased from Sigma-Aldrich (Saint-Louis, MO, USA). SSP, a mixture of Chestnut and Quebracho wood extracts (60/40 w/w) was supplied by SilvaTeam S.p.a. (San Michele Mondovì, Italy). Tannin percentage of SSP powder (92.4%) was obtained by gravimetric analysis of vegetable tanning agents by using the filter Freiberg-Hide powder method (Küntzel, 1954).

### Sperm thawing

Boar frozen semen (0.5 mL straws) was purchased from a commercial company (Inseme S.P.A., Modena, Italy). Three different animals (1 ejaculate each) were used. For each experimental repetition, three straws from the same ejaculate were thawed in a water bath at 37 °C for 30 sec and subsequently pooled and diluted with three volumes of Beltsville Thawing Solution (BTS). Only thawed samples with sperm viability higher than 40%, as evaluated by SYBR14/PI test (see below) were used. Thawed semen was immediately divided in the following experimental groups: CTR (control: without SSP addition) and SSP (addition of 5, 10 and 20 µg/mL SSP; SSP5, SSP10, SSP20 respectively). After incubation for 1 h at 37 °C, each semen group was divided in three aliquots: one was used for sperm parameters analysis, one for IVF trials and the last one for the immunolocalization of tyrosine-phosphorylated proteins either at the end of incubation in BTS or after an additional incubation for 1h in IVF medium at 39 °C 5%CO_2_ (see below IVF section).

### Sperm motility

Sperm motility was measured by means of a computer-assisted sperm analysis system (CASA, Hamilton Thorne, IVOS Ver. 12); the standard boar setup was used (60 frame per sec; 45 frames captured; min contrast 49; min cell size 6 pixels; progressive cells: VAP 20.1 μm/s; straightness percentage 75; static cell cut-off: VAP 20 μm/s, VSL 5 μm/s). Approximately one thousand cells at 30 × 10^6^ sperm/mL were evaluated for each sample using a fixed-height Leja Chamber SC 20-01-04-B (Leja, The Netherlands). Parameters assessed were percentages of total motile spermatozoa (TM), percentages of progressively motile spermatozoa (PM), curvilinear velocity (VCL μm/s), average path velocity (VAP μm/s), straight line velocity (VSL μm/s), percentages of straightness (STR) and linearity (LIN), average lateral head displacement (ALH μm) and beat cross frequency (BCF Hz).

Together with global sample analysis, individual sperm tracks were assessed and VCL, VAP, VSL, STR, LIN, ALH and BCF were recorded for each motile spermatozoon.

### Flow cytometry analysis

Information about flow cytometry analyses is reported taking into account the recommendations of the International Society for Advancement of Cytometry (Lee et al., 2008). Flow cytometry analyses were conducted to evaluate sperm viability, acrosome integrity, mitochondrial function and lipid peroxidation. In each assay, sperm concentration was adjusted to 1 × 10^6^ spermatozoa/mL in a final volume of 0.5 mL BTS, and spermatozoa were then stained with the appropriate combinations of fluorochromes, following the protocols described below. Samples were evaluated through a FACSCalibur flow cytometer (Becton Dickinson, Milan, Italy) equipped with a 488 nm argon-ion laser. Emission measurements were made by means of three different filters: 530/30 band-pass (green/FL-1), 585/42 band-pass (orange/FL-2) and >670 long pass (far red/FL3) filters. Data were acquired using the BD CellQuest Pro software ver. 6.0 (Becton Dickinson).

Signals were logarithmically amplified and photomultiplier settings were adjusted to each particular staining method. FL1 was used to detect green fluorescence from SYBR14, fluorescein isothiocyanate (FITC)-conjugated Pisum Sativum Agglutinin (PSA), low mitochondrial membrane potential (JC1 negative), and BODIPY 581/591, whereas FL2 was used to detect orange fluorescence from high mitochondrial membrane potential (JC1 positive) and FL3 was used to detect orange-red fluorescence from Propidium Iodide (PI).

Side scatter height (SS-H) and forward scatter height (FS-H) were recorded in logarithmic mode (in FS vs SS dot plots) and sperm population was positively gated based on FS and SS while other events were gated out. A minimum of 10.000 sperm events were evaluated per replicate.

In FITC-conjugated PSA flow cytometry assessment, percentages of non-DNA–containing particles (alien particles, f) were determined to avoid an overestimation of sperm particles in the first quadrant (q1) as described by Petrunkina et al. (2010), according to the following formula:

d1=q1−f100−f×100 (1)

where q1 is the percentage of non-stained spermatozoa after correction.

### Sperm membrane integrity

Sperm viability was assessed by checking the membrane integrity using two separate fluorochromes SYBR-14 and PI (LIVE/DEAD Sperm Viability Kit; Molecular Probes, Invitrogen, Milan, Italy). SYBR-14 is a membrane-permeable dye, which stains the head of viable spermatozoa in green, while PI is a membrane-impermeable dye that only penetrates through disrupted plasma membrane, staining the sperm heads of non-viable cells in red. Sperm samples were diluted with BTS to a concentration of 1 × 10^6^ spermatozoa/mL and aliquots of 500 μL were stained with 5 μL SYBR-14 working solution (final concentration: 100 nM) and with 2.5 μL of PI (final concentration: 12 μM) for 10 min at 37°C in darkness. Viable spermatozoa exhibited a positive staining for SYBR-14 and negative staining for PI (SYBR-14+/PI-). Single-stained samples were used for setting the voltage gain for FL1 and FL3 photomultipliers.

### Acrosome integrity

Sperm acrosome intactness was assessed by Pisum Sativum Agglutinin (PSA) conjugated with fluorescein isothiocyanate (FITC) (2.5 mg/mL stock solution; 0.5 mg/mL working solution) coupled with Propidium Iodide (2.4 mM stock solution). Sperm samples were diluted with BTS to a concentration of 1 × 10^6^ spermatozoa/mL and aliquots of 500 μL were stained with 10 μL FITC-PSA (final concentration: 10 μg/mL) and with 3 μL PI (final concentration: 14 μM) for 10 min at 37 °C in darkness. Four different sperm subpopulations were distinguished: a) viable acrosome-intact spermatozoa were those cells that did not stain with either FITC-PSA or PI and appeared in the lower left quadrant of FL1 vs. FL3 plots; b) viable spermatozoa with disrupted acrosome stained only in green with FITC-PSA and were found in the lower right panel; c) non-viable spermatozoa with intact acrosome stained with PI only and appeared in the upper left quadrant; and d) non-viable spermatozoa with disrupted acrosomes were found in the upper right quadrant and stained positively with both stains.

### Mitochondrial membrane potential

5,5′,6,6′-tetrachloro-1,1′,3,3′-tetraethylbenzimidazolyl carbocyanine iodide (JC-1) was used to evaluate mitochondrial membrane potential. When it comes in contact with mitochondria with high membrane potential, JC-1 forms multimers (known as J-aggregates) and emits orange fluorescence at 590 nm, which is detected by FL-2 photomultiplier. In contrast, when mitochondria have low membrane potential, JC-1 maintains its monomeric form (M-band) and emits green fluorescence at 530 nm, which is detected by FL-1 photomultiplier.

Sperm samples were diluted with BTS to a concentration of 1 × 10^6^ spermatozoa/mL and aliquots of 500 μL were stained with 5 μL JC1 (at a final concentration of 1 μg/mL) and 3 μL of PI (at a final concentration of 14 μM); samples were successively incubated at 37 °C for 30 min in the dark. PI positive cells were gated out in a FL-1/FL-3 dot plot; PI negative cells were gated and analysed in a FL-1/FL-2 plot. High mitochondrial membrane potential cells (HMMP) stained orange (higher FL-2) and low mitochondrial membrane potential cells (LMMP) stained green (higher FL-1).

### Lipid peroxidation

Bodipy 581/591 (Molecular Probes Eugene, CA, USA) stock solution was prepared diluting 1 mg of the molecule in 1980 μL DMSO. For analysis, sperm samples were diluted with BTS to a concentration of 1 × 10^6^ spermatozoa/mL; aliquots of 500 μL were centrifuged at 900× g for 2 min at room temperature; the supernatant was discarded, and sperm pellet resuspended with 492 μL BTS and stained with 5 μL BODIPY stock solution (final concentration 0.01 μg/mL and 3 μL of PI (at a final concentration of 14 μM). Cells were incubated for 30 min at 37 °C in the darkness and subsequently analysed.

As no separate sub-populations in FL1-FL3 plots were detectable, a relative fluorescence quantification method was used, as described by Bucci et al. (2018). Briefly, the instrument was set with 10 references of the same ejaculate of frozen-thawed boar semen and the mean FL1 signal was registered. For each analysis, one sample of the same reference was used to set the voltage and gain of the instrument to get the same reference value; subsequently the experimental samples were run.

### Protein tyrosine phosphorylation immunostaining

The study of sperm tyrosine phosphorylation was made before and after capacitation for 1h in BO medium. Briefly, an aliquot of spermatozoa incubated for 1 h at 37 °C from each experimental group was fixed as below described, while another aliquot was washed twice in BO medium, resuspended in the same medium at a final concentration of 30 × 10^6^ spermatozoa/mL and incubated 1 h in a humidified atmosphere of 5% CO2 in air at 39 °C. At the end of incubation samples were fixed as below described. Three different animals (1 ejaculate each) were used and each experiment was repeated twice (n = 6).Aliquots of sperm cells from the different experimental groups were spotted onto poly-l-lysine-coated slides and fixed with methanol at -20 °C for 15 min and with acetone for 30 s. The slides were then washed with PBS and blocked with 10% (v/v) FCS (Gibco) in PBS (blocking solution) for at least 30 min. Antibody dilutions were performed in blocking solution. Monoclonal anti-phosphotyrosine antibody (clone 4G10, Merck Millipore, Darmstadt, Germany) was added at the dilution 1:150. Incubation was carried out overnight at 4 °C. After extensive washing with PBS, sperm cells were incubated with a sheep-anti-mouse FITC-conjugated secondary antibody (BioFX Laboratories, Maryland, USA) for 1 h in the dark. Slides were washed with PBS and mounted with Vectashield mounting medium with PI (Vector Laboratories). Control slides were treated similarly with the omission of primary antiserum. Spermatozoa were evaluated with the above described epifluorescence microscope. Each sample was analysed by counting at least 200 cells in order to evaluate the different positivity patterns by the same observer, blinded to the experimental group of each sample. Four different patterns were considered on the basis of what assessed by Bucci et al. (2012):

A: positivity in the Equatorial Subsegment (EqSS) and acrosome;
B: positivity in the acrosome, EqSS and principal piece of the tail;
C: positivity in the tail and (not constant) in the EqSS;
NEG: spermatozoa with no positive signal.

### Oocytes collection and *in vitro* maturation (IVM)

Ovaries were collected from pre‐pubertal gilts at a local slaughterhouse and transported (in 0.9% w/v NaCl solution) to the laboratory within 2 h. Cumulus‐oocyte complexes (COCs) were aspirated from antral follicles, 3-6 mm in diameter, with a 18‐gauge needle fixed to a 10‐mL disposable syringe. Intact COCs were selected under a stereomicroscope and only COCs with more than three layers of compact cumulus cells and with uniform cytoplasm were transferred into a petri dish (35 mm, Nunclon, Denmark) prefilled with 2 mL of modified PBS supplemented with 0.4% BSA. After three washes in NCSU 37 (Petters and Wells, 1993) supplemented with 5.0 μg/mL insulin, 1 mM glutamine, 0.57 mM cysteine, 10 ng/mL epidermal growth factor (EGF), 50 μM β-mercaptoethanol and 10% porcine follicular fluid (IVM medium), groups of 50 COCs were transferred to a Nunc 4-well multidish containing 500 μL of the same medium per well and cultured at 39 °C in a humidified atmosphere of 5% CO_2_ in air. For the first 22 h of *in vitro* maturation the medium was supplemented with 1.0 mM db-cAMP, 10 IU/mL, eCG (Folligon, Intervet, Boxmeer, The Netherlands) and 10 IU/mL hCG (Corulon, Intervet). For the last 22 h COCs were transferred to fresh maturation medium (Funahashi et al., 1997) without db-cAMP and eCG/hCG. At the end of the maturation period the oocytes were denuded by gentle repeated pipetting.

### *In vitro* fertilization (IVF)

For *in vitro* fertilization trials aliquots of sperm suspensions from the different experimental groups were washed twice in Brackett and Oliphant's medium (BO)(Brackett and Oliphant, 1975) supplemented with 12% heat inactivated fetal calf serum (Gibco, Invitrogen, Italy)(FCS) and 0.7 mg/mL caffeine (BO medium) and then resuspended in the same medium at a final concentration of 0.5 × 10^6^ spz/mL. Five hundred µL of the sperm suspensions were placed to each well of Nunc 4-well multidish. Then groups of 50 *in vitro* matured oocytes were transferred to each well and, after 1 h of co-culture, oocytes were transferred to fresh BO medium and cultured for 17-18 h. The oocytes were then mounted on microscope slides, fixed in acetic acid/ethanol (1:3; v/v) for 24 h and stained with Lacmoid. Oocytes were observed under a phase-contrast microscope and parameters evaluated were: penetration rate (number of oocytes penetrated/total inseminated), monospermy rate (number of oocytes containing only one sperm head-male pronucleus/total fertilized) and total efficiency (number of oocytes containing only one sperm head–male pronucleus/total inseminated). Degenerated and immature oocytes were not counted.

### Statistical analysis

Statistical analyses were performed using R version 3.4.0 (2017-04-21) (Copyright ^©^ 2017, The R Foundation for Statistical Computing) Values are expressed as mean ± standard deviation (SD), unless otherwise specified and level of significance was at P≤0.05. Motility and post thawing parameters assessed by flow cytometry and tyrosine phosphorylation data expressed as percentages were transformed with arcsine square root. Subsequently all variables (both motility and post thawing parameters) were tested for normality and homogeneity of variances through Shapiro-Wilk and Levene tests. One-way ANOVA and Tukey post hoc test were used to assess differences between treatments. As for IVF trials, the variables (i.e. penetration rates and monospermy) were analysed using a general linear model with binomial distribution and a Tukey post-hoc test was subsequently run to determine differences between treatments.

### Ethics approval and consent to participate

The semen used in this study was purchased from a commercial company.

## Results

The addition of SSP at the concentrations of 5, 10, 20 µg/mL to thawed sperm for 1 h did not exert any significant effect on sperm motility parameters (TM, PM, VCL, VAP, VSL, ALH, BCF, STR, LIN) viability, acrosome integrity, mitochondrial functionality and lipid peroxidation (Tables 1 and 2).

**Table 1:** Effects of SSP (5, 10, 20 µg/mL) supplementation to thawed boar sperm on sperm motility parameters.

**Group**	**TM**	**PM**	**VCL**	**VAP**	**VSL**	**ALH**	**BCF**	**STR**	**LIN**
CTR	40.5±6.5	16.3±4.3	116.1±12.6	65.0±5.9	51.1±7.3	4.7±0.6	37.8±2.5	74.8±7.3	45.5±7.8
SSP 5	40.3±7.4	16.7±4.1	118.8±10.6	65.4±4.4	50.6±5.1	4.5±0.5	38.3±2.1	75.3±4.9	44.5±5.9
SSP 10	37.8±6.6	15.5±4.0	118.2±13.0	65.4±4.9	50.9±4.9	4.6±0.5	38.4±1.7	74.8±6.0	44.7±6.3
SSP 20	38.0±8.3	16.2±4.3	113.6±13.2	62.0±3.9	48.5±4.5	4.5±0.4	38.3±3.0	75.6±5.7	44.3±6.1

TM: total motility (%); PM: progressive motility (%); VCL: curvilinear velocity (µm/sec); VAP: average path velocity (µm/sec); VSL: straight line velocity (µm/sec); ALH: amplitude of lateral head displacement (µm); BCF: beat cross frequency (Hz); STR: straightness (%); LIN: linearity (%). CTR: control; SSP: Silvafeed SP. Values are expressed as the mean ± SD (standard deviation) of six replicates (three boars). One-way ANOVA and Tukey post hoc test were used to assess differences between treatments.

**Table 2:** Effects of SSP (5, 10, 20 µg/mL) supplementation to thawed boar sperm on sperm parameters.

**Group**	**Sperm viability %**	**Acrosome integrity %**	**HMMP %**	**Lipid peroxidation**
CTR	46.4±6.1	49.0±6.5	77.9±11.5	44.5±9.2
SSP 5	47.4±6.7	49.7±7.8	78.9±12.4	44.3±10.2
SSP 10	48.9±4.4	50.1±7.3	80.6±9.3	45.9±8.5
SSP 20	48.1±7.9	52.7±5.2	82.5±12.8	44.7±6.5

CTR: control; SSP: Silvafeed SP. Values are expressed as the mean ± SD (standard deviation) of six replicates (three boars). HMMP: percentage of living cells with high mitocondrial membrane potential. Lipid peroxidation: lipid peroxidation of living cells membrane (mean fluorescence intensity of BODIPY 581/591, arbitrary units). One-way ANOVA and Tukey post hoc test were used to assess differences between treatments.

Tyrosine phosphorylated protein immunolocalization, used as capacitation parameter, did not change after one hour of incubation in BTS in the presence of SSP (5, 10, 20 μg/mL) compared to control.

After washing and incubating spermatozoa of the different experimental groups for 1h in BO medium, the overall percentage of cells displaying A pattern (non capacitated cells) significantly decreased (P<0.01) and the overall percentage of capacitated spermatozoa showing patter B increased (P<0.01), while no significant difference was observed in the percentages of the spermatozoa showing the different phosphorylation patterns of the tyrosine among the experimental groups (Table 3).

**Table 3:** Effects of SSP (5, 10, 20 µg/mL) supplementation during 1h post-thaw incubation on tyrosine-phosphorylation of spermatozoa fixed immediately (A) or at the end of a further incubation of 1h in capacitating condition (B).

**A**				
**Group**	**Pattern A (%)**	**Pattern B (%)**	**Pattern C (%)**	**NEG (%)**
CTR	79.5±10.6	0.7±0.9	0.1±0.2	19.6±10.3
SSP5	73.1±13.1	1.0±1.2	0.0±0.0	25.9±13.4
SSP10	75.1±12.4	1.3±1.2	0.1±0.2	23.5±12.2
SSP20	74.7±11.4	1.1±1.0	0.1±0.2	24.0±11.7
**B**				
**Group**	Pattern A (%)*	Pattern B (%)*	**Pattern C (%)** ^*^	**NEG (%)**
CTR	45.5±20.6	21.1±9.2	7.2±5.5	26.1±13.4
SSP5	44.4±20.4	22.1±10.1	7.4±3.8	26.1±12.6
SSP10	44.1±21.8	20.2±9.4	7.1±3.5	28.7±15.4
SSP20	43.8±21.1	20.4±8.2	7.1±4.1	28.7±14.5

Pattern A (non capacitated cells): positivity in the EqSS and acrosome. Pattern B (capacitated cells) (positivity in the acrosome, EqSS and principal piece of the tail). Pattern C (acrosome reacted cells): positivity in the tail and (not constant) in the EqSS. NEG: spermatozoa with no positive signal. CTR: control; SSP: Silvafeed SP. One-way ANOVA test was used to assess differences between time and treatments.

* Indicates significant differences (P< 0.01) in the overall data between 1h post-thaw incubation and after a further 1h incubation in capacitating condition.

The IVF results are shown in Table 4. Oocytes inseminated with thawed spermatozoa pretreated with all the different SSP concentrations tested presented a significantly (P < 0.01) increase in penetration rate compared to CTR group (SSP5 60.9±8.9%, SSP10 65.9±3.5%, SSP20 70.7± 8.7%, CTR 42.5± 6.8%). In addition, 5 µg/mL SSP supplementation exerted a positive (P<0.05) effect on the total efficiency of fertilization as compared to CTR (39.3±7.2% vs 28.3± 4.2% respectively) (Table 4).

**Table 4:** Effects of SSP (5, 10, 20 µg/mL) supplementation to thawed boar sperm on IVF parameters.

**Group**	**Number of oocytes**	**Penetration rate %**	**Monospermy rate %**	**Total efficiency of fertilization %**
CTR	379	42.5±6.8^a^	68.3±15.3^a^	28.3±4.2^a^
SSP 5	237	60.9±8.9^b^	65.1±12.0^a^	39.3±7.2^b^
SSP 10	263	65.9±3.5^b^	55.0±9.7^ab^	36.3±6.9^ab^
SSP 20	244	70.7±8.7^b^	42.6±13.7^b^	29.4±7.7^ab^

Penetration rate (number of fertilized oocytes / number of inseminated oocytes). Monospermy rate (number of oocytes containing only one sperm head–male pronucleus / number of penetrated oocytes expressed as a percentage). Total efficiency of fertilization (number of monospermic oocytes / number of inseminated oocytes expressed as a percentage). CTR: control; SSP: Silvafeed SP. Values are expressed as the mean ± SD (standard deviation) of at least six replicates (three boars). The variables were analysed using a general linear model with binomial distribution and a Tukey post-hoc test was subsequently run to determine differences between treatments. Different letters indicate significant difference in column between treatments.

## Discussion

Freezing and thawing of boar spermatozoa cause considerable cell damage leading to severe reductions in farrowing rates and litter size after AI. Boar spermatozoa are especially susceptible to cold shock (White, 1993) as their membranes have a relatively high proportion of PUFAs that renders spermatozoa highly susceptible to lipid peroxidation due to excessive generation of ROS. Furthermore, not all boars have ejaculates that present the same freezability and there is a significant variability between and within-ejaculates (Peña et al., 2006; Yeste, 2016). For this reason, boars and their ejaculates are classified into “good” (GFE) or “poor” (PFE) freezers on the basis of their post-thaw sperm survival and motility (Casas et al., 2009).

In recent years supplementation of antioxidants in the cryopreservation media has given interesting and promising results in different species included pig (Yeste, 2016). Antioxidants can reduce the oxidative damage by scavenging ROS or inhibiting the generation of ROS.

The present work aimed at limiting the negative effects of ROS generation by adding to thawing medium SSP, a natural extract rich in polyphenols and, in particular, tannins, molecules known to possess antioxidant properties (Koleckar et al., 2008). After thawing, sperm quality, in terms of motility, viability, acrosome integrity, mitochondrial functionality and lipid peroxidation was assessed via flow cytometry. The addition of different doses of SSP to thawed sperm did not induce any significant effects on sperm motility and viability compared to control as already reported by other Authors who worked in the same experimental conditions but used different natural antioxidant components like epigallo-catechin-3-gallate (Gadani et al., 2017) and Astragalus polysaccharide (Weng et al., 2018), while total and progressive motility were negatively affected by resveratrol (Bucci et al., 2018). In our study, acrosome integrity, mitochondrial activity and lipid peroxidation were not influenced by SSP addition indicating that this natural extract has no effect on these parameters at the concentrations studied.

The main finding emerging from this study is the beneficial effect of SSP supplementation on *in vitro* fertilization parameters. In fact, oocytes inseminated with thawed spermatozoa pretreated with all the different SSP concentrations showed a higher penetration rate (P<0.01). In addition, 5 µg/mL SSP exerted a positive effect (P<0.05) on the total efficiency of fertilization as it increased penetration rate without increasing the polyspermy. This result is of particular interest taking into account the well-known reduced ability of *in vitro* matured pig oocyte to efficiently block polyspermy (Funahashi, 2003). Interestingly, a previous study showed that two tannin relatives, tannic acid and ellagic acid, decrease the incidence of polyspermy in porcine oocytes blocking the hyaluronidase activity of sperm; however, in that research tannins were added to BO medium during the fertilization (Tokeshi et al., 2007) while in our study SSP was no more present during gamete coincubation.

In recent studies we showed similar beneficial effects on IVF parameters of two natural polyphenols with antioxidant activity (resveratrol and epigallo-catechin-3-gallate) when supplemented, alone or in combination, to boar sperm thawing medium (Gadani et al., 2017; Bucci et al., 2018). The positive effect of SSP, similarly to that previously recorded for resveratrol and epigallo-catechin-3-gallate, was exerted during IVF and therefore after washing away the tested molecules: SSP was left with semen for one hour after thawing and then spermatozoa, after washing and therefore removing SSP, were resuspended in IVF medium. Based on these results we may hypothesize that the protective effect during thawing can lead to positive effects on sperm function that are maintained even when SSP is no more present.

The mechanism by which SSP increases penetration and efficiency rate (at a concentration of 5 µg/mL) remains to be clarified as none of the different SSP concentrations tested (5, 10, 20 µg/mL) significantly modified the sperm parameters assessed.

In order to try to explain the mechanism by which SSP influences IVF parameters, the degree of sperm capacitation was assessed by the immunolocalization of phosphorylated tyrosine residues. After one hour of post-thawing incubation in the presence of SSP, no significant differences were observed between the different groups. Even following *in vitro* induction of sperm capacitation by semen washing and incubation in capacitating medium, no significant differences were observed in the percentage of cells showing tyrosine-phosphorylation pattern of capacitated cells among the different experimental groups. Recently Spinaci et al. (2018) have instead shown that an ethanolic extract of tannins from Quercus Robur is able to modulate the degree of sperm capacitation with a significant increase in the spermatozoa displaying the capacitated pattern. It has to be taken into account that in that study the ethanolic tannin extract was present during incubation in a capacitating medium while in this study SSP was no longer present.

Therefore, since no significant effects were found on the analyzed sperm parameters, further studies are necessary to clarify the mechanism by which the post-thawing incubation with SSP is able to increase *in vitro* fertilization ability. Moreover, the constituents of SSP, natural extract rich in polyphenols, have not been identified; therefore which of them is/are responsible for the biological activity remains to be established.

## Conclusions

The data concerning the positive action of SSP on the parameters of *in vitro* fertilization are of particular interest in that these results suggest that the positive effects of the extract are maintained even after its removal. These results encourage the use of this additive in the thawing medium since post-thawing fertility is a limit for the use of large-scale frozen semen (Didion et al., 2013; Knox, 2015, 2016; Yeste et al., 2017). Further researches are needed to verify whether the positive effect on IVF is exerted and confirmed also *in vivo*. The selection of “good freezers” boars is a crucial strategy for the application of frozen-thawed boar semen on a large scale; the males we used for IVF trials were chosen because of their good semen quality after thawing and their ability to fertilize oocytes *in vitro*. It cannot be excluded that SSP may improve the fertilizing ability also of semen from bad freezer boars and this possibility remains to be verified. Further studies are necessary to investigate the possible positive effect of SSP addition to commercial thawing solutions during porcine AI with frozen-thawed semen since post-thawing fertility is a limit for the large-scale use of boar frozen semen. Lastly, further researches are also necessary to confirm antimicrobial property of this natural extract that it could be an alternative to the use of antibiotics in the swine AI doses.
